# Integrative Analysis Using Module-Guided Random Forests Reveals Correlated Genetic Factors Related to Mouse Weight

**DOI:** 10.1371/journal.pcbi.1002956

**Published:** 2013-03-07

**Authors:** Zheng Chen, Weixiong Zhang

**Affiliations:** 1 Department of Computer Science and Engineering, Washington University, St. Louis, Missouri, United States of America; 2 Department of Genetics, Washington University School of Medicine, St. Louis, Missouri, United States of America; Harvard Medical School, United States of America

## Abstract

Complex traits such as obesity are manifestations of intricate interactions of multiple genetic factors. However, such relationships are difficult to identify. Thanks to the recent advance in high-throughput technology, a large amount of data has been collected for various complex traits, including obesity. These data often measure different biological aspects of the traits of interest, including genotypic variations at the DNA level and gene expression alterations at the RNA level. Integration of such heterogeneous data provides promising opportunities to understand the genetic components and possibly genetic architecture of complex traits. In this paper, we propose a machine learning based method, *module-guided Random Forest*s (mgRF), to integrate genotypic and gene expression data to investigate genetic factors and molecular mechanism underlying complex traits. mgRF is an augmented Random Forests method enhanced by a network analysis for identifying multiple correlated variables of different types. We applied mgRF to genetic markers and gene expression data from a cohort of F2 female mouse intercross. mgRF outperformed several existing methods in our extensive comparison. Our new approach has an improved performance when combining both genotypic and gene expression data compared to using either one of the two types of data alone. The resulting predictive variables identified by mgRF provide information of perturbed pathways that are related to body weight. More importantly, the results uncovered intricate interactions among genetic markers and genes that have been overlooked if only one type of data was examined. Our results shed light on genetic mechanisms of obesity and our approach provides a promising complementary framework to the “genetics of gene expression” analysis for integrating genotypic and gene expression information for analyzing complex traits.

## Author Summary

Obesity has become a perilous global epidemic that can lead to complex diseases, such as diabetes and cardiovascular diseases. Much effort has been devoted to the studies of the genetic mechanisms that pillow the manifestation of obesity. Although a large quantity of experimental data has been accumulated lately using high-throughput techniques, our understanding of genetic mechanisms of obesity is still limited. The proposed method is motivated to address three critical issues that have impeded the existing methods. The first is the curse of dimensionality in selecting a subset of genetic elements related to the traits of interest from a large number of candidates. The second is genetic multiplicity underlying non-Mendelian traits, in which multiple genes are in interplay. The third issue is the integration of data from multiple sources in light of genetic multiplicity and curse of dimensionality. Here, we propose a new method, which augments the Random Forests method with a network-based analysis, to integrate genotypic and gene expression information and identify correlated multiple genetic elements underlying mouse weight. Our results shed light on complex genetic interactions underlying obesity, which can form viable hypotheses worthy of further investigation.

## Introduction

Most complex traits such as obesity involve a diverse set of genes, intricate interplay among them and subtle interaction between genetic and environment factors. One of the first steps toward a systematic understanding of the genetic basis of a complex trait is the identification of causal genetic elements, e.g. genes, genetic markers and/or single nucleotide polymorphisms (SNPs), whose variations are responsible for the traits. The objective of this challenging task is two-fold: effectively identifying a subset of genetic elements out of a large pool of candidates whose patterns are characteristic of a trait of interest, and accurately predicting the phenotype with a model that accommodate interactions among selected genetic elements. Despite recent advances in high-throughput technologies that have produced an enormous amount of biological data, heterogeneous data types, non-linear relationships among genes and complex phenotypes have made this task difficult.

Although conventional linkage analyses and association studies as well as the latest genome-wide association studies (GWAS) have produced a fruitful collection of genomic susceptibility loci for a variety of complex traits and diseases [1], [2], they have mainly been able to detect genetic elements of marginal effects while failed to respect epistatic interactions [3], [4]; as a result, they have a low power for predicting phenotypes [5]. As an intermediate between genotype and phenotype, gene expression has been proven to be a rich and valuable source of information complementary to genotype information for dissecting complex traits. On one extreme using gene expression data alone, classifiers or regressors have been built to predict disease types or stages with only a small number of disease-related genes [6]–[8]. By integrating information of genetics and gene expression, genetics of gene expression-based approaches [9]–[11] and network-based approaches [12]–[14] have been independently developed and applied to identify genes related to complex traits. Recently a few machine learning based methods have been proposed to integrate both genotype and gene expression data to not only identify relevant genes, but also predict phenotypes based on selected genes. Ruderfer et al. [15] adopted a SVM classifier to predict drug responses (i.e., sensitivity or resistance) in yeast. They showed that using both data of transcripts and genetic markers can improve prediction accuracy compared with using either transcripts or genetic markers alone. Based on the elastic net regularized regression [16], Chen et al. [17] developed Camelot to predict quantitative response (i.e. growth yield) of yeast to 94 drugs using genotype and gene expression data collected from drug-free conditions from yeast segregants. Compared with the work by Ruderfer et al. [15], Camelot was able to make accurate quantitative prediction on various drug treatments as opposed to dichotomic classes of drug response. Camelot also emphasized greatly on causal inference by incorporating *a priori* knowledge and adopting post statistic tests to select only handful genetic makers and expression transcripts as phenotype predictors. For example, for predicting the hydrogen peroxide response, only a single gene, *DHH1*, passed their pre-filtering criteria and was then used to construct the final prediction model. Although appropriate for downstream experimental validation as Camelot always make the most conservative choices, it remains unknown whether its filtering steps could indeed help improve prediction accuracy and whether it would otherwise prevent further novel discovery besides the factors known to have large marginal effects.

Random Forests (RF) [18], an ensemble of classification or regression trees, has recently been successfully applied in various biological studies [19]–[23]. RF has many desirable characteristics that make it well suited for integrating both genotypic and gene expression information. It is well adapted for variable selection for high-dimensional data with competing prediction accuracy compared to the state-of-the-art machine learning techniques. RF is able to accommodate categorical (e.g. genotype) and continuous (e.g. gene expression) data. It can be used when the number of variables substantially exceeds the number of observations (e.g. thousands of SNP markers and probes of gene expression versus a few hundred samples of subject) [19]–[23]. Moreover, RF supports possible interactions among variables [4], which is critical for systems-biology studies where interplays between genetic (e.g. epistatically interacting SNPs) and gene expression (e.g. coactivator/corepressor) must be taken into consideration. While promising, however, conventional RF algorithms have several drawbacks that limit their success on large biological problems. Firstly, even though RF allows possible interactions among variables, it does not incorporate possible correlation among variables; even worse, with correlated variables, it suffers from biases introduced in measuring variable importance (VI) [24], [25], which can result in incorrect or misleading variable rankings. Secondly, RF's prediction accuracy may decline significantly when the proportion of truly informative variables among all variables is small [26].

In this paper, we develop a new method, called module-guided Random Forests (mgRF), to integrate genotypic and gene expression information to understand and possibly dissect complex relationships among different genetic elements underlying complex traits. mgRF combines the method of conventional RF and a network-based analysis to remedy the two aforementioned drawbacks of conventional RF by exploiting structural relationships, extracted from the network analysis, among different types of variables. As a test and application, we applied mgRF to the data of genetic markers and gene expression from a cohort of F2 female mouse intercross to examine its performance and demonstrate its ability to identify genetic elements that contribute to mouse weight, many of which were missed by the conventional RF algorithm. mgRF outperformed the state-of-the-art methods that combine information from multiple biological sources with more accurate predictions. Furthermore, using mgRF we investigated the interactions among multiple genetic elements underlying mouse weight. Statistically significant interactions of SNP-to-SNP, gene-to-gene, and SNP-to-gene identified by mgRF revealed genetic elements and their significant association underlying mouse weight. The results demonstrated a great expectation of mgRF as a complementary framework to “genetics of gene expression” analysis for dissecting genetic mechanism of complex traits, such as obesity.

## Results

### Overview of the mgRF method

**Figure 1:**
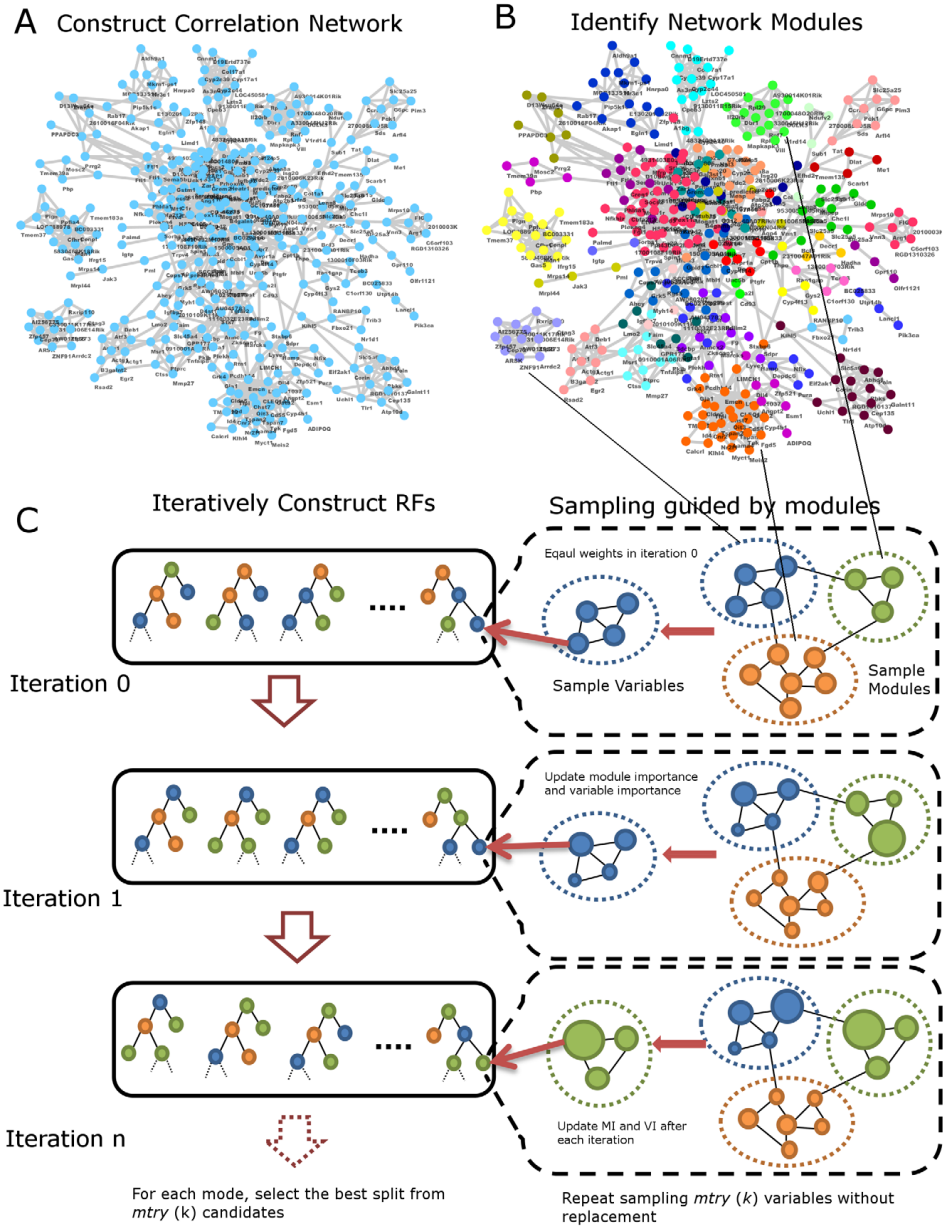
Flow chart of mgRF algorithm. (**A**) A subgraph of a constructed correlation network where a node indicates a variable and an edge represents the correlation between two variables. (**B**) Identification of network modules using HQcut, where different colors encode different modules. (**C**) Iterative construction of RFs. The panels on the left show multiple iterations of RFs and the panels on the right illustrate the sampling scheme in each node during of tree construction. *mtry* (*k*) candidate variables are sampled using a *two-stage candidate variable sampling* procedure, where a subset of modules (e.g. the blue, green, orange modules) is first sampled and then one representative variable from each module is selected. In the first iteration (iteration 0), all modules and variables have the same weights. At the end of one iteration, module and variable importance are re-estimated. In the figure, the importance of variables is encoded as the size of node. Then modules and variables are sampled by their corresponding weights using *modified weighted sampling*. The best splitting variable and value at each node in the tree in the left panel are selected from *mtry* (*k*) candidate variables.

In mgRF our main objectives are to capture intrinsic structures of variable (genetic element) correlation and/or interaction and to incorporate such information in the RF framework to predict a complex phenotype. The major steps of mgRF algorithm, outlined in Figure 1, consist of the identification of variable modules from a variable correlation network (Figures 1A and 1B) and an iterative RFs construction process (Figure 1C). In the first step we construct a correlation network and identify modules in the network to group highly-correlated variables, which may be in different types, using a network clustering method such as HQCut [27]–[29]. In the second step of mgRF, we iteratively construct a series of RFs guided by the previously identified network modules. Instead of randomly sampling variables in each node of regression tree, we adopt a *two-stage candidate variable sampling* procedure, where we first select a subset of modules and then choose one representative variable for each of the selected modules (right panels in Figure 1C) to correct the bias of variable importance while incorporating the variable association information. Except the first RF construction, we use a *modified weighted sampling* to improve the prediction accuracy by prioritizing informative variables among a large pool of variables. A key element of mgRF is to correct possible bias of variable importance measure and improve the performance of RF for high-dimensional data. This is done in part by introducing a module importance (MI) to each network module identified. Initially all MI and variable importance (VI) are set to 0 so that in the first iteration the sampling of modules and variables is un-weighted. After each iteration of RF, new MIs and VIs are re-estimated (not accumulated). The values of MIs and VIs can typically converge within a small number of iterations, where little change can be observed between the last and the second to the last estimations. The final output of mgRF is an ensemble of trees as a model for future analysis and its corrected VIs (cVIs) and MIs for variable and module ranking. Details and parameter selections of the mgRF algorithm are described in Materials and Methods.

### Combining genotypic and expression data can better predict mouse weight

**Figure 2:**
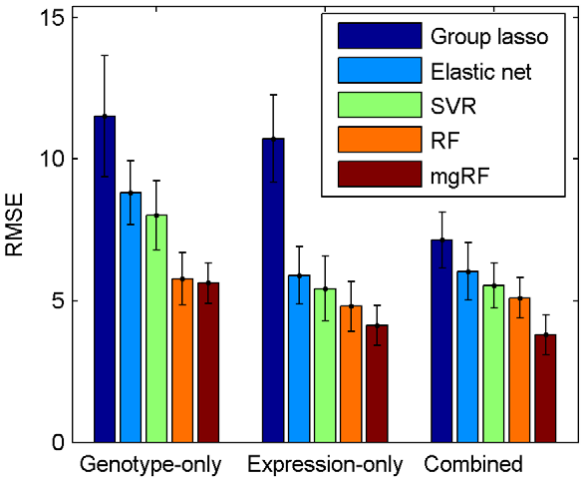
Performance comparison of Group lasso, Elastic net, Support Vector Regressor (SVR), conventional RF, and mgRF. Average RMSEs of these methods in a 10-fold cross-validation on the mouse weight dataset. Even though the standard deviation (shown as error bar) of RMSEs among 10 trials of 10-fold cross-validation is relatively high due to the small number of samples, if we compare the RMSEs of different methods using the same set of training samples, the improvement is evident (mgRF vs conventional RF, one-tail paired t-test *p*-value<3.83×10^−16^).

To investigate the benefits of integrating genotypic and gene expression data, we examined the performance of different models on the genotypic and gene expression data of a cohort of F2 mouse intercross in a three-way comparison: (1) using only data of genetic markers (genotype-only), (2) using only data of gene expression (expression-only), and (3) using both genotypic and expression data (combined). We first compared mgRF with group lasso [30], elastic net [16], SVR-RFE [8], and the conventional RF algorithm (see Text S1) in terms of the weight regression error (Root-Mean-Square Error or RMSE) in all three types of data using 10 trials of 10-fold cross-validation. The average cross-validation RMSEs of the methods compared are shown in Figure 2. mgRF achieved the smallest average error compared to all the other competing methods in all types of data. Since we assessed each model using the same training and test data in each fold of the cross-validation, we can compute the paired t-test of RMSEs to evaluate the significance of the results. As shown in Table S1, the RMSEs of all the other methods are significantly larger (p<2.52×10^−13^) than that of mgRF. Furthermore, the running time of mgRF is slightly less than the conventional RF with better prediction accuracy (Table S2). It is noteworthy to mention that SVR-RFE, RF and mgRF outperformed the linear models, group lasso and elastic net, suggesting the benefits of incorporating non-linearity between variables and the mouse weight response.

**Figure 3:**
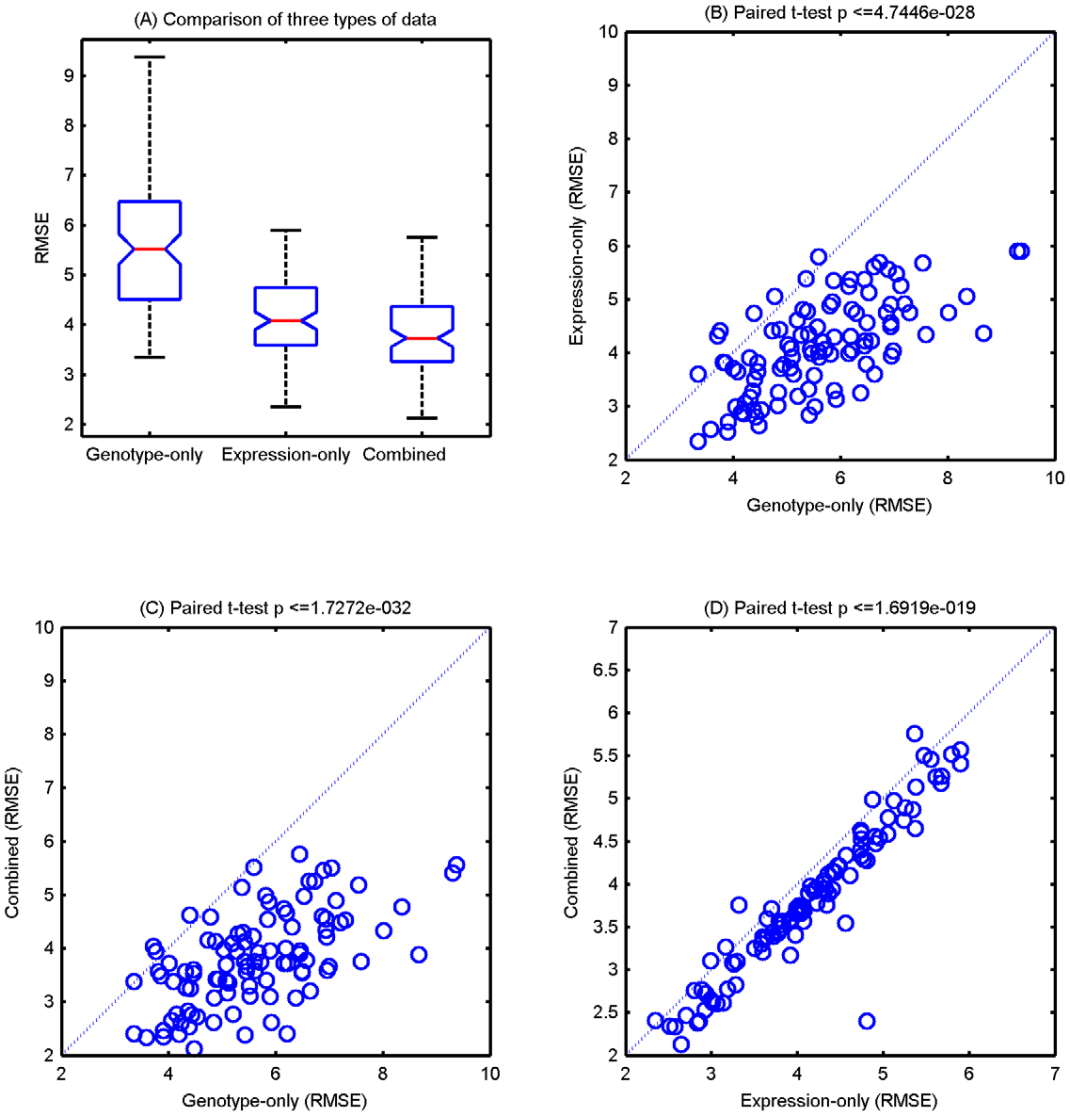
Summary of prediction errors of mgRF using three types of data. (**A**) Boxplot of prediction errors in root-mean-square error (RMSE) in 10 trials of 10-folds cross-validation. Scatter plots of (**B**) genotype-only (x-axis) vs. expression-only (y-axis), (**C**) genotype-only vs. combined, and (**D**) expression-only vs. combined. The dashed diagonal lines (x = y) indicate points of equal RMSE. Given two vectors, the *p*-value of one-tail paired *t*-test evaluate if the distribution of one vector (100 RMSE values for 10 trials of 10-fold cross validation) is statistically smaller than the other one.

In particular, we examined the RMSEs of mouse weight using mgRF. The boxplot of prediction errors on the three data types are shown in Figure 3A. The combined data have the smallest error (RMSE = 3.80 and R^2^ = 0.604), followed by the expression-only data (RMSE = 4.13, and R^2^ = 0.534), while the genotypic-only data have the highest error rate (RMSE = 5.62 and R^2^ = 0.137). Thus using either the genotypic or gene expression data alone is less effective than using the combined data. Although the standard error of RMSEs of different trials (quartile bar in Figure 3A) is relatively large comparing to the difference of mean RMSE between with and without genotypic data, there is a substantial improvement in pairwise comparisons using the same training samples (Figures 3B to 3D). The two-dimensional co-ordinates of point in each of these plots indicate the RMSEs of mgRF trained with the same set of samples but with different data types. In Figures 3B and 3C, most of the points appear under the reference diagonal line, which confirms that both expression-only and combined data achieved better performance in a single fold than the genotype-only data (paired one-tail t-test *p*-value≤4.7446×10^−28^ and ≤1.7272×10^−32^, respectively). This was probably because in general the linkage signal of genetic markers is weak (LOD score <4), while gene expression is more closely related to the phenotype than genotypes. Furthermore, mgRF using both genotypic and gene expression data outperforms using expression-only data in more than 90% of the trials (Figure 3D, paired one-tail t-test p-value≤1.691×10^−19^), showing that combining genotypic and gene expression data can indeed improve the prediction power and suggesting that information of gene expression plays a role in bridging the gap between genotype variations and complex traits.

### Genetic elements identified by mgRF reveal perturbed pathways related to mouse weight

The mgRF method used corrected variable importance (cVI) and module importance (MI) to identify variables and groups of variables that influence the trait of body weight. MIs were computed for network modules identified by the HQcut algorithm [27], [29], [31]. To assess and illustrate mgRF's ability for correcting the bias of variable importance, we evaluated different regression models regarding their abilities for recovering the true variable importance associated with the known data-generating pattern in a simulation study (see Text S2). mgRF was able to accurately recover the known pattern of variables' importance and the VI measure of mgRF was more stable than the other methods in all simulations, as discussed in Text S2.

**Figure 4:**
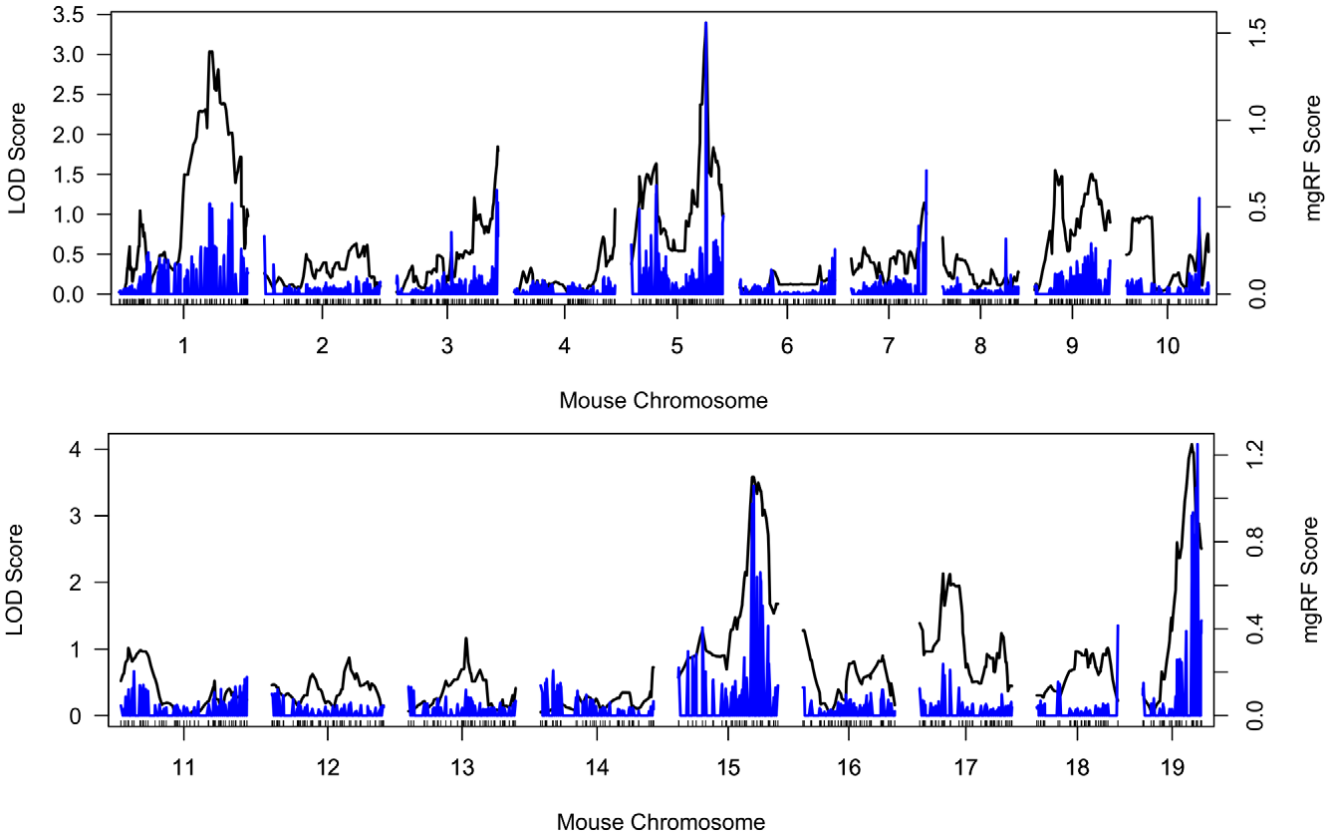
LOD scores from QTL mapping versus VI scores from mgRF. The black curves represent the LOD scores of a single marker genome scan in conventional QTL analysis. The blue bars represent the cVI scores of genetic markers output by mgRF.

When applied to the mouse weight data from a cohort of 132 samples and compared with modules identified by topological overlap matrix based methods [12], [32], HQcut produced much smaller modules, allowing only highly-correlated variables to be clustered in a module (Figure S1). HQcut identified 146, 1036 and 1187 network modules (see module structures in Table S3, S4, S5) in the genotype-only, expression-only and combined data, respectively. As expected, SNPs in one module were generally in linkage disequilibrium. Genes in one module were co-expressed and potentially functionally related. There were SNPs and genes assigned to the same module in the combined data set due to the large correlation values among those gene expression and SNPs. The top-ranked genetic markers and genes in the combined data largely overlapped with those identified by genotype-only and expression-only data types indicating the stability of mgRF in terms of variable ranking. Here we reported the top-ranked modules of genetic markers and genes in Table 1 and 2. Among these top-ranked SNPs (Table 1), rs3662726 (Chromosome 5, 123 Mb) is near Gofm2 (gonadal fat mass 2) QTL which has been reported to confer increased fat mass in female mouse [33]. We also examined the LOD scores of SNPs using the traditional QTL mapping. Several “hotspot” QTLs on Chromosomes 1, 3, 5, 7, 10, 15 and 19 were partially overlapped with the top-ranked markers by mgRF (Figure 4). Table S6 lists all the top-ranked SNPs. Note that several markers with low LOD scores were assigned relative high cVIs, suggesting that a SNP with low marginal effect can be identified by mgRF because of their interaction with other SNPs, which may contribute to the variation of body weight.

**Table 1:** Representative significant SNPs in the top 10 modules identified by mgRF in the combined dataset.

Marker ID	UCSC ID	SNP Physical Location	mgRF cVI	QTL LOD	Candidate eQTL Genes
p46143	rs3677464	Chr 1, 142 Mb	0.519253	3.03	Ugt1a9, Mogat1, Igfbp2
p45505	rs3659655	Chr 1, 177 Mb	0.520485	2.01	Grpel2, Copa, Cd244
p45693	rs3714147	Chr 3, 159 Mb	0.598267	1.62	Cnn3, A430056A10Rik
p45975	rs3672859	Chr 5, 47 Mb	0.624049	1.63	Bmp2k, Tgfbr2, Slc2a9
p45558	rs3662726	Chr 5, 123 Mb	1.560222	3.4	Bmp1k, Spp1, Mtf2, Tslpr, Gpr109b
p44866	rs3714636	Chr 7, 137 Mb	0.710581	1	Asc
p44776	rs3680872	Chr 10, 117 Mb	0.552147	0.84	Lzp-s, Kitl
p44593	rs3667621	Chr 15, 80 Mb	1.057362	3.58	Dlgap2, Rac2, Ang2, Ncf4, Ccl4
p45916	mCV23069037	Chr 19, 52 Mb	0.934463	3.95	Atad1, Lzts2, Cyp2c40, Tjp2
p44699	rs3663566	Chr 19, 56 Mb	1.248977	2.88	Lzts, Cyp2c40

**Table 2:** Representative significant genes in the top 10 modules identified by mgRF in the combined dataset.*

Module ID	Symbol	cVI (%)	Description
32	Igfbp2	10.94238	insulin-like growth factor binding protein 2
	Cyp2c37	9.654316	cytochrome P450, family 2. subfamily c, polypeptide 37
	Fmo3	9.41088	flavin containing monooxygenase 3
	Reep5	8.20887	receptor accessory protein 5
	C7orf24	7.4338	chromosome 7 open reading frame 24
	Gpld1	6.813601	glycosylphosphatidylinositol specific phospholipase D1
189	Mogat1	11.84315	monoacylglycerol O-acyltransferase 1
	MGC137458	11.67155	hypothetical LOC541113
	Raet1d	9.386647	retinoic acid early transcript delta
244	Pdia5	6.81388	protein disulfide isomerase associated 5
	1300018K11Rik	5.812176	-
	Heatr1_predicted	5.385251	HEAT repeat containing 1 (predicted)
307	Slc43a1	6.548385	solute carrier family 43, member 1
	Acsm1	4.96683	acyl-CoA synthetase medium-chain family member 1
	BC029214	3.816577	cDNA sequence BC029214
150	Angpt2	6.038289	angiopoietin 2
	Zfp521	5.677051	zinc finger protein 521
68	Avpr1a	5.682891	arginine vasopressin receptor 1A
	Cd93	3.815527	CD93 antigen
468	Serpinf2	4.113287	serine (or cysteine) peptidase inhibitor, clade F, member 2

(*Note: Top genes ranked by mgRF in the expression-only data were the same as the one above with slightly different cVIs).

It is important to note that there was little overlap among the 100 top-ranked genes from group lasso, elastic net, SVR-RFE, the conventional RF algorithm, and mgRF (Figure S2A). The lack of consensus indicated that these algorithms identified their own top-ranked genes based on different (unspecified) assumptions on the given data and target models to be learned. Introduction of such assumptions seemed to be inevitable because of the lack of sufficient knowledge of the problem at hand and different objectives that these methods were devised to achieve. Nevertheless, all these methods strived to select predictive variables (genetic factors). On top of finding individual predictive genetic factors, mgRF was particularly designed to identify such predictive genetic factors whose association might contribute more significantly than individual variables at the module level because it propagated the contribution of individual variables to highly correlated neighbors, rather than fully focusing on individual genes. Table S7 lists the top-ranked genes related to mouse weight from mgRF. Among these top-ranked genes (Table 2), monoacylglycerol O-acyltrasferase 1 (*Mogat1*, cVI = 11.84) in module 189 has been previously identified to be located within Chromosome 1 obesity QTL interval near D1Mit215. Within this QTL interval on Chromosome 1, insulin-like growth factor binding protein 2 (*Igfbp2*, cVI = 10.94) has expression levels in liver negatively correlated with mesenteric fat pad weights [34]. *Igfbp2* (appeared in module 32) has also been shown to prevent diet-induced obesity and insulin resistance in mice on overexpression [35]. In particular, module 32 contained *Cyp2c37* (cVI = 9.65), *C7orf24* (cVI = 7.43) and *Gpld1* (cVI = 6.54), which were not among the top 100 ranked genes from any of the other methods compared, probably due to their correlation with *Igfbp2*. *Raet1d* (cVI = 9.38) in module 189 was also not identified by the competing methods (Table S8) probably due to its correlation with *Mogat1*. Remarkably, *Cyp2c37* has been previously recognized as being associated with fat mass [10] and *Gpld1* had been shown to be associated with the level of adiponectin, a hormone secreted from adipose tissue which is negatively correlated with obesity [36]. It is viable to hypothesize that other genes identified by mgRF, which were neglected by the other methods, may potentially contribute to mouse weight variation. To further assess the biological significance of the genes identified by mgRF, we conducted a Gene Ontology (GO) enrichment analysis (see Materials and Methods) on the top-ranked genes from the methods that were compared. mgRF identified more enriched biological processes than the other methods (Table S9). In particular, genes identified by mgRF were enriched with many obesity-related processes, such as regulation of lipid storage (*p* = 7.17×10^−06^), positive regulation of cholesterol storage (*p* = 1.07×10^−05^), regulation of growth (*p* = 0.000575), and cellular response to cholesterol (*p* = 0.00396). In contrast, the enriched biological processes provided by the other methods were less significant and less biologically meaningful (Tables S5B to S5E).

**Figure 5:**
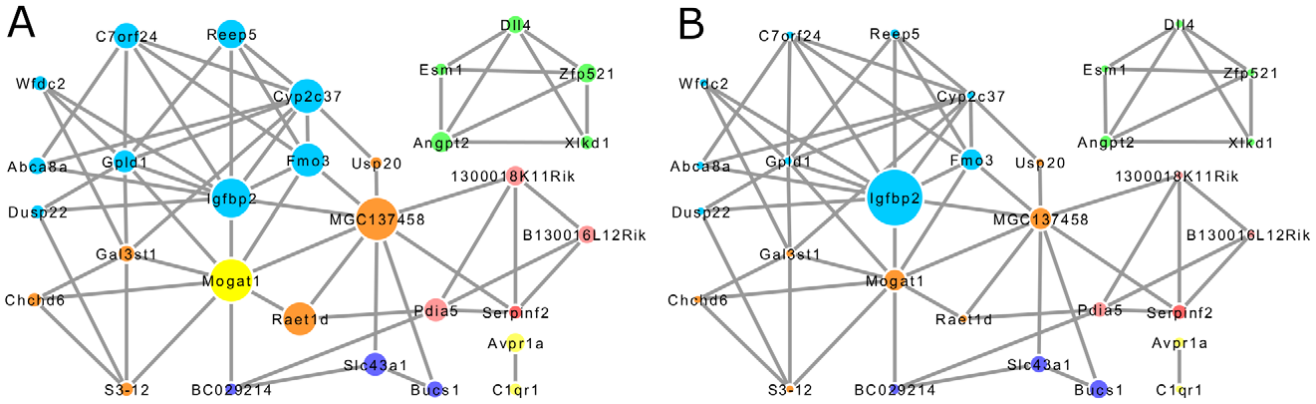
Network structure of 30 top ranked genes from mgRF. (**A**) The size of nodes represents the relative cVIs from mgRF. (**B**) The size of nodes represents the relative VIs from the conventional RF algorithm.

As an example, Figure 5 shows a sub-network of some top-ranked genes from mgRF to compare the variable importance measures in mgRF and the conventional RF. The size of nodes represents relative cVIs from mgRF in Figure 5A and represents relative VIs from conventional RF in Figure 5B. In the conventional RF algorithm, one variable, *Igfbp2*, has a larger importance than others. As a result, the importance of *Igfbp2* overshadows several other correlated variables such as *Fmo3*, *Cyp2c37*, and *Raet1d*, which may in fact be equally important as *Igfbp2*. In mgRF, several genes with the highest cVI, such as *Mogat1*, *MGC137458*, and *Igfbp2*, which are known to be critical to body weight, were also hub nodes in the network with many edges. It was consistent with our previous studies on the importance of hub genes in the co-expression network [37].

### Significant interactions among multiple genetic elements revealed by mgRF

Although the corrected variable importance (cVI) from mgRF quantifies the contribution of a genetic factor to the prediction power, it does not indicate whether the contribution is from the genetic factor alone or from its interaction between or association with other factors. One advantage of the RF method is its ability to incorporate variable interactions, which mgRF inherited. We devised a systematic statistical test (see Materials and Methods) to assess the significance of gene-to-gene, SNP-to-SNP, and SNP-to-gene interactions revealed by mgRF. To examine the biological relevance of genes identified in gene-to-gene interactions in the mouse weight data, we first tested the functional enrichment among 160 unique genes from the top 100 most significant pairs of interactions (Table S10). Interestingly, these genes were enriched with metabolic processes such as isoprenoid metabolic process (*p* = 0.00675), drug metabolic process (*p* = 0.00841), and terpenoid metabolic process (*p* = 0.00117), indicating obesity-related interaction among the identified genes. In particular, the pair of *Avpr1a* and *Igfbp2* is one of the most significant interactions (*p* = 4.15×10^−07^), both of which are also among the most predictive genes. However, only 11 (∼7%) of the 160 unique genes from the significant gene-to-gene interactions were overlapped with the top 100 most predictive genes identified by cVI (Figure S2B). This suggested that our interaction test could indeed identify genes that were less significant when examined individually. For example, macrophage receptor with collagenous structure (*Marco*) was observed to interact with many other phenotype-related genes (e.g. *Dhrs4*, *Cyp2d22*, and *Pdia5*), even though its own cVI was relatively low. Among the top-ranked SNP-to-SNP interactions, we found significant interactions between SNPs on Chromosome 5 (123 Mb) and Chromosome 19 (51 Mb) (*p* = 3.34×10^−11^), on Chromosome 2 (96 Mb) and Chromosome 9 (61 Mb) (*p* = 8.97×10^−7^), and on Chromosome 1 (41 Mb) and Chromosome 15 (62 Mb) (*p* = 2.94×10^−06^, Table S11). The most significant interaction was between p45558 (Chromosome 5, 123 Mb) and p44890 (Chromosome 19, 51 Mb). Cis-eQTLs analysis [12] indicated that *Bmp2*, a key regulator of adipogenesis, was a candidate gene of p45558, and *Cyp2c40*, known to be presented in Fatty acid metabolism, was a candidate gene of p44890. For SNP-to-gene interactions (Table S12), there was little overlapping between genes involved in SNP-to-gene interactions and genes involved in gene-to-gene interactions (Figure S2B). Of particular interest was the interaction between SNP p45334 (Chromosome 1, 77 Mb) and gene *Ehhadh*, where *Mogat1* is one of the candidate eQTL genes of p45334 and *Ehhadh* is annotated in the fatty acid metabolism pathway. We compared the top 50 unique SNPs involved in SNP-to-gene interactions with top SNPs ranked by cVIs. More than 20 of them were among the top-50 most predictive SNPs. On the other hand, only two genes involved in top 100 SNP-to-gene interactions were among the top 100 most predictive genes. We hypothesized that the most predictive SNPs did not interact with the most predictive genes because the information contained in these SNPs was redundant to these predictive genes, which usually were the expression traits of the corresponding predictive SNPs. In turn, by combining less predictive gene and marker profiles introduced extra information into the system and indeed improved the prediction accuracy. Genes involved in such interactions might reveal additional perturbed pathways underlying the trait of body weight.

## Discussion

A systems biology approach is necessary to dissect complex traits, such as obesity, and understand relationships among various genetic factors. Combing heterogeneous data from multiple sources will become increasingly important to model a large quantity of data and interpret results. In this paper, we proposed a novel approach that integrates the method of Random Forests and a network analysis to incorporate genotypic and gene expression data for revealing genetic factors and their interaction or association that are characteristic of complex traits. To overcome the curse of dimensionality, mgRF enhanced conventional RF with the module structure of a correlation network and the weighted sampling procedure. As a result, it successfully identified a small subset of both predictive and biological meaningful genes and genetic markers out of thousands of candidates. Meanwhile, mgRF was able to model the complex associations and possibly interactions between heterogeneous variables, which lead to interesting findings that can shed some lights on solving the genetic multiplicity problem underlying complex traits.

To rectify the bias in ranking correlated variables in conventional RF, simple but effective strategies such as grouping correlated variables prior to model fitting [38], [39] can be applied, where cluster centroids obtained from a hierarchical clustering could be used as supergenes to fit classification/regression models. Compared with Tolosi and Lengaue's work [39], the major differences and novelty of mgRF are three folds. (1) We maintained the original feature space in RF models so that the importance of individual variables can be estimated. (2) Instead of using a simple hierarchical clustering, we adopted the network modeling method HQCut, which is able to automatically and accurately determine the number of modules (clusters) in the network. (3) We further utilized the learnt MI and VI to guide a weighted sampling of variables. Compared with other regression models, such as elastic net and support vector regressor (SVR), mgRF naturally handles different types of variables in that the splitting points of continuous (or ordinal) variables preserve the order information, which, however, is disregarded in categorical variables. On the contrary, elastic net and SVR treat categorical variables as continuous ones, consequently imposing ordered information, which is related to how the categories should be encoded. There is a popular variable importance measure, permutation VI [18], which measures the increase of out-of-bag prediction error with the variable to be measured being permutated. However, the permutation VI suffers from several shortcomings for large problems. It requires an excessive computation time, since each variable needs to be permuted dozens of times to ensure statistical stability. In addition, when the baseline prediction error is large, there is little chance for permutation to make a prediction worse, which leads to an uniformly low VI [25]. More critically, it is still subject to the bias of correlated variables [40], [41]. Even though this problem can be corrected [24], [42], [43], the incurred computation time of additional permutation for a solution will make the excessive computation cost prohibitive for large application.

Using a BxH F2 mouse intercross data set, we showed that the proposed algorithm was effective on not only reducing prediction error, but also identifying a subset of genetic markers and genes that are associated with the trait of body weight. By integrating genotypic and gene expression data, mgRF achieved a lower prediction error compared to using either type of data alone. These results support the idea that gene expression plays an intermediate bridging role between genotypic variations and a phenotype. Genotypic data alone are insufficient for accurately predicting the body weight due to their relative weak effects, while gene expression data bridge the gap between genotypic variants and a phenotype as gene expression can be intermediate traits of multiple genetic markers. Besides the annotated body weight relevant genetic elements, such as QTL rs3662726, genes *Mogat1*, *Igfbp2*, and *Cyp2c37*, mgRF provided valuable hypotheses on putative, novel genetic elements and their interactions that are potentially important for body weight and obesity. In particular, the top-ranked SNPs and genes, which have similar levels of importance but are lack of known annotations, are excellent candidates for further validations.

A key feature of mgRF is that it exploited splitting variables to incorporate non-linear interactions of variables into the model and to identify intriguing associations within and across two types of data. The proposed statistical test for variable associations aimed at extracting biological relevant markers and genes that might have been overlooked by individual variable importance ranking. The results of mgRF showed that several known obesity-related genes and loci were associate or even interacted with each other and genes that were strongly associated were indeed related to the traits of obesity and/or body weight, as these genes were enriched with biological processes on metabolisms. More importantly, the results revealed that many genetic elements, which have not been indicated previously to be associated with the traits, interacted with obesity-related genes and their associations may contribute more significantly to the traits than associations between genes that were known to be related to obesity. In addition, the results also included significant pairs of genes even though the predictive scores of individual genes whose predictive scores were insignificant. These results suggested that more obesity related genetic factors remain to be discovered and mgRF is potentially an enabling method for identifying genetic factors whose significance would not be appreciated unless their associations or interactions were taken into consideration.

Another key advantage of RF is that at each splitting point, it only considers *mtry* (*k*) candidate variables for splitting, usually *k<<m*, where *m* is the number of variables. The time complexity of mgRF is the same as the conventional RF, in the order of 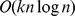. In practice, mgRF is usually more efficient than the conventional RF, thanks to the *two-stage candidate variable sampling* and the *modified weighted sampling*. In particular, the *mtry* (*k*) of mgRF is proportional to the number of modules instead of the actual number of variables. In our experiments, the average training time for mgRF was below 3 minutes for our C++ implementation on a desktop machine with an Intel Duo core 2.53 GHz CPU and 4 G memory (see Table S2 for running time comparison). The most time consuming part of the mgRF framework is the network construction and module finding using HQCut, which took around 20 minutes on the same machine. For larger gene expression data, a common practice is to pre-filtering low variance genes to ∼10,000 most varying genes. For large SNPs data, to reduce the time of network construction and clustering, LD-pruning can be utilized to approximate the SNPs' module structure.

Compared with conventional “genetics of gene expression” analysis, mgRF provided a complementary means to incorporate the knowledge of inherent structure of genetic elements. mgRF can also readily be applied to more than two types of data and can be efficient on large-scale applications as it can be easily parallelized to utilize the growing cloud computing environments. While mgRF includes statistical tests to identify significant pair-wise interactions among variables, there is amble room for identifying higher order interactions within the framework of variable importance.

## Materials and Methods

### Data preparation and preprocessing

The BxH mouse weight dataset consists of both categorical and continuous variables: gene expressions of 7,441 most varying genes and genotypes of 1,065 genetic markers that exhibit variation between two parental strains. The problem can be formulated as a regression problem of predicting the body weights of 132 F2 intercrossed female mice using these two types of variables. The detailed information regarding the experiment and data collection is in Ghazalpour et al. [12].

### Correlation network construction and identification of network modules

Given a high-dimensional dataset with multiple types of variables, we adopted a previously developed network construction and clustering method HQCut [27]–[29] to identify the intrinsic structures of variables. HQCut is able to group variables into clusters, i.e. network modules, using a parameter-free spectral clustering-based method to optimize a network modularity function [44]. HQCut has been applied to analyze complex human disease such as Alzheimer's disease [28], [37]. Pearson's correlation was used to compute the correlation between two continuous variables (e.g. gene expression). The correlation between two categorical variables (e.g. genetic markers) was defined as their normalized Mutual Information. For the correlation between a categorical variable and a continuous variable, we first discretized continuous variable *X* into three categories, given by
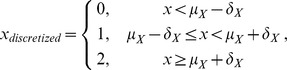
where *μ_X_* is the mean and *δ_X_* is the standard deviation of *X*. We then calculated the correlation of the discretized variable with the categorical variable using Mutual Information. Given the correlations of all pairs of variables, we constructed the correlation network using the same method described in [27]–[29]. For two variables to be connected in the network, their correlation needs to satisfy at least one of the following criteria: (1) the correlation is greater than 0.5 and one of the variable is ranked among the top 5 most correlated variables of the other variable; (2) the correlation is greater than 0.8 and one of the variables is ranked among the top 50 most correlated variables of the other. These two criteria ensure a sparse weighted network structure while maintaining both local (via rank-based threshold) and global (via value-based threshold) properties [29]. Since different types of variables might have correlation values in different scale, the ranking threshold is independent of each pair of data types. For example, if genotypic and gene expression data are provided, above criteria will be separately applied on SNP-to-SNP, gene-to-gene, and SNP-to-gene (or the other direction) respectively. HQcut is then applied to identify the optimal partitioning that cluster genes into non-overlapping modules and automatically determine the number of modules based on the modularity function [44].

### Module-guided Random Forests

The basic building blocks of RF for regression problems are the regression trees [45], which recursively partitioning the dataset into two subsets based on a specific variable among all variables to minimize the squared loss. RF is an ensemble of regression trees, where each tree is built using a set of bootstrap samples, which is a subset of the original sample. At each splitting (or internal) node of a tree only *mtry* (*k*) small randomly selected variables (or attributes) are evaluated. The overall prediction of a forest is the majority vote or the average over the predictions from all individual trees. In a bagging iteration, approximately one third of the observations are not used. These unused observations, the so-called out-of-bag (OOB) sample, can be used to estimate the generalization error. In general, three parameters are needed to be determined in the conventional RF algorithm and mgRF: *ntrees* (*n*), the number of trees in the forest, *mtry* (*k*), the number of candidate variables that each node considers to find the best split, and *nodesize* (*s*), the minimum size of sample in a node where no further splitting is needed. Since a large *ntrees* usually stabilizes variable importance measures, we set it to a large number (*ntrees* = 1000) in our experiments. We set *mtry = m/3*, one third of the number of variables as recommended for regression problems. We set *nodesize* to 3 as opposed to the recommended 5 because the sample size of our datasets is usually small (<200). Our preliminary experiments have shown that the RF and mgRF are insensitive to parameter choices.

To reduce previously mentioned bias on the variable importance and to incorporate priori knowledge of variables' structure, we adopted a *two-stage candidate variable sampling* procedure: we first selected a subset of modules, each of which captured a set of correlated variables, and then chose one representative variable from each module to form the candidate splitting variables for each node in RF. Given the module structure of a correlation network, we sampled a subset of modules as candidate modules, from which candidate splitting variables are selected in the second stage. By using the *two-stage candidate variable sampling*, we could significantly reduce the number of variables to be evaluated in each split as the number of modules was typically much smaller than the best *mtry* parameter in the original RF. We further defined the module importance (MI) of a module *m_i_* to be the sum of VIs of its member variables, 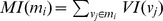. MI of a particular module summarized the contribution of all its member variables. We further defined the corrected variable importance (cVI) to estimate the importance of individual variables. The cVI was defined as a weighted sum of VIs of all its connected neighbors within the same module.
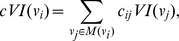
where *M*(*v_i_*) is the module that *v_i_* belongs to and *c_ij_* is the measure of correlation between variables *v_i_* and *v_j_*.

We further enhanced mgRF by recursively building a series of RFs, where the selection of candidate splitting variables was not only guided by the module structure as in the *two-stage candidate variable sampling* procedure, but also guided by MIs and VIs that generated from previous RF. Except the first RF, where both modules and variables were uniformly chosen, the construction of successive RFs follows a *modified weighted sampling* scheme, which combined both uniform and weighted sampling, to favor informative variables. Take the sampling of modules as an example, given a set *S* of weighted modules, a subset *S_1_* of size *N_1_* was randomly chosen from *S* without replacement, where the probability of selecting *S_1_* was proportional to the weights. We then uniformly selected *N_2_* modules from *S_1_* to form a smaller set *S_2_*, which was the set of final candidate modules. The same procedure was applied to selecting variables from each module. The choices of *N_1_* and *N_2_* depended on the data analyzed. In principle, *N_1_* should be large enough to cover truly relevant objects (i.e. informative variables) and *N_2_* should be small enough to allow diversity of splitting variables. In the current study we set *N_1_* = *N/3*, where *N* is the size of modules (or variables in one module) and performed weighted sampling based on MIs (or VIs) for module (or variable) sampling. In module sampling, we set 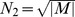, where |*M*| was the number of modules, because we assumed that there should be multiple latent factors contributing to the trait variable. In variable sampling we let *N_2_* = 1 as previously discussed to ensure the unbiased property of MI. Note that the variable sampling was weighted on VIs instead of cVIs during each run of RFs. In other words, we only corrected VIs generated in the last iteration because VIs of variables within a module are good estimation of their relative importance, which is sufficient for sampling a representative variable from a given module.

### Gene Ontology enrichment analysis

We downloaded the latest MGI mouse Gene Ontology annotations from Gene Ontology consortium [46]. Given the 7,441 most varying genes as the background, and a list of genes to be test, each GO biological processes term was assigned a *p*-value to quantify the significance of gene-term enrichment using the Fisher's exact test.

### Statistical analysis of variable interactions

Regression trees are well suited for modeling non-linear effects such as epistatic interactions because of its conditional splitting method used. We expect that some variables in a regression tree are more likely to be split on when the tree has already been split on a corresponding interacting variable. We derived a simple but effective statistical test to assess the significance of such interactions based on the Fisher's exact test. In particular, given two variables *u* and *v*, we counted the number of times they appeared in an ensemble of *N* trees as *n* and *m*, respectively. Under the null hypothesis of *u* and *v* being independent, the number of times, *k*, that they both appears in the same tree should follow the hypergeometric distribution,
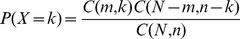
where *C*(*x,y*) is a binomial coefficient that choosing *y* from *x*. The *p*-value from the test is computed by summing over the probability of right-tail extremes, where *k* is set to (*k*, min(*m*, *n*)). Note that our definition is slightly different from the one implemented in the conventional RF [18] for classification, where a test based on the Gini importance is applied. In our test, we used the counts of variables being chosen to cope with continuous traits. In our experiment, we used N = 6000 to achieve a stable ranking of interactions.

## Supporting Information

Figure S1 Distribution of the sizes of modules produced by HQCut on mouse weight data. HQcut produced relatively small modules that cluster variables with high degrees of correlation. In particular more than 80% of modules contain less than 20 variables.
(TIFF)

Figure S2 (A) Venn diagram of the 100 top-ranked genes identified by Group lasso (blue), Elastic net (yellow), SVR-RFE (orange), Conventional RF (green), and mgRF (purple). (B) Venn diagram of top ranked genes from gene ranking and genetic element interaction analysis from mgRF. In (B), blue circle represents the top 100 most predictive genes ranked by cVIs. Orange circle represents 160 unique genes ranked in the top 100 most significant gene-to-gene interactions. Green circle represents 100 genes in the top-ranked SNP-to-gene interactions. Most of the genes involved in significant interactions are not individually predictive of mouse weight.
(TIFF)

Figure S3 The regression errors of RF and mgRF with different clustering methods and number of clusters. The average RMSEs of mgRF and RF are shown as straight lines because their performance is invariant to the number of clusters. The average RMSEs of other method with respect to a specific number of clusters are shown as (1) Random clustering: dash-dotted line with square markers, (2) K-means: dash line with star markers and (3) Hierarchical clustering: dash-dotted line with cross marker.
(TIFF)

Figure S4 The regression errors of RF and mgRF with respect to different *mtry* (*k*) values. The average RMSEs of conventional RF in genotype-only, expression-only, and combined dataset are plotted with dash line and star markers. The average RMSEs of mgRF in genotype-only, expression-only, and combined dataset are plotted with solid line and square markers.
(TIFF)

Figure S5 Variable importance identified by various methods with different cardinality of G_1_ in the simulation dataset. The importance values are normalized to percentage in (**A**) Group lasso, (**B**) Elastic net, (**C**) SVR-RFE, (**D**) conventional RF, and (**E**) mgRF. In mgRF, importance values are shown in cVI. The gray dotted lines indicate the grouping of variables. The first 110 variables are relevant variables from construction. For G_1_ and G_2_, the importance values of 10 uniformly distributed variables are plotted from each group.
(TIFF)

Table S1 p-values of paired t-test when comparing RMSE of mgRF with RMSE of other methods (Group lasso, Elastic Net, SVR, and RF).
(XLSX)

Table S2 Average running time (seconds/fold) of 10-fold cross-validation on mouse weight dataset.
(XLSX)

Table S3 Network module structure in the SNP-only data.
(XLSX)

Table S4 Network module structure in the Expression-only data.
(XLSX)

Table S5 Network module structure in the combined data.
(XLSX)

Table S6 Top-ranked SNPs sorted by cVI of mgRF.
(XLSX)

Table S7 Top-ranked Genes sorted by cVI of mgRF.
(XLSX)

Table S8 List of 100 top-ranked genes identified by Group lasso, Elastic net, SVR-RFE, and conventional RF.
(XLSX)

Table S9 Gene Ontology enrichment analysis of top-ranked genes (*p*-value<0.01) identified by mgRF, group lasso, elastic net, SVR-RFE, and conventional RF.
(XLSX)

Table S10 Top 500 significant pair-wise gene-to-gene interactions.
(XLSX)

Table S11 Top 500 significant pair-wise SNP-to-SNP interactions.
(XLSX)

Table S12 Top 500 significant pair-wise SNP-to-gene interactions.
(XLSX)

Table S13 Stability score of variable importance in different methods (Group lasso, Elastic net, SVR, RF, and mgRF) with various size of |*G_1_*| in the simulation dataset.
(XLSX)

Text S1 The supplementary text to give the introduction on other regression methods compared with mgRF.
(PDF)

Text S2 The supplementary text to give the methods and results for several comparisons studies.
(PDF)
