# Supplementary figures and images for: Integrative Analysis Using Module-Guided Random Forests Reveals Correlated Genetic Factors Related to Mouse Weight
Source: PLoS Comput Biol. 2013 Mar 7;9(3):e1002956. doi: 10.1371/journal.pcbi.1002956 (PMC3591263; doi:10.1371/journal.pcbi.1002956)

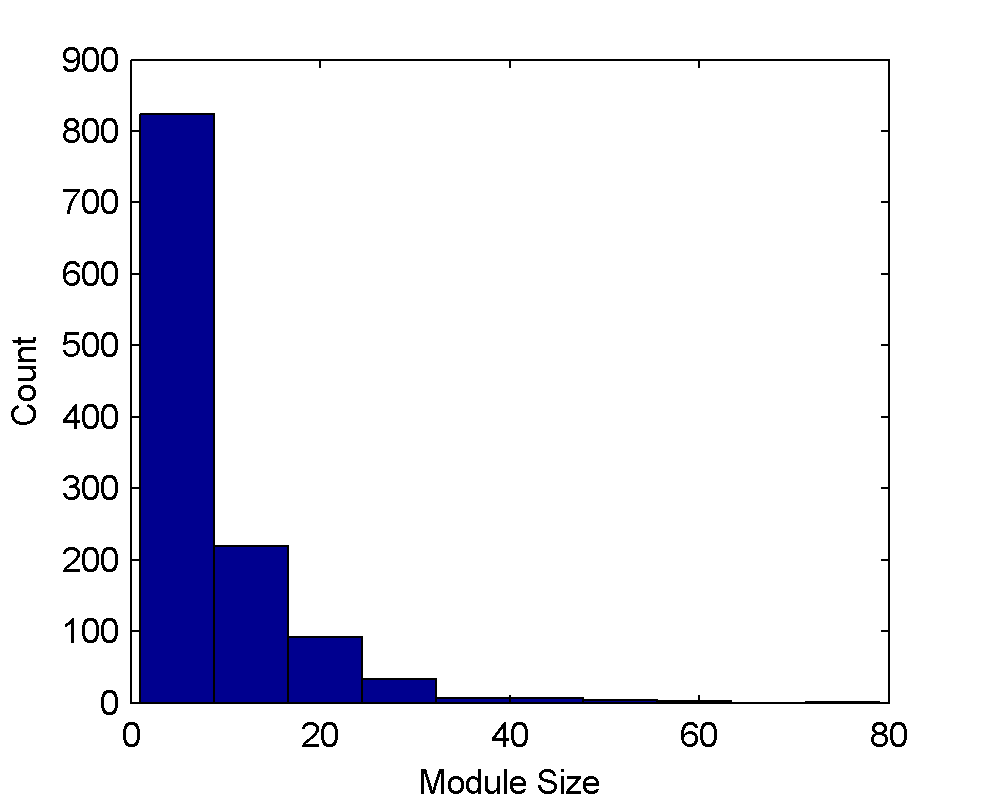

Supplement: Figure S1 — Distribution of the sizes of modules produced by HQCut on mouse weight data. HQcut produced relatively small modules that cluster variables with high degrees of correlation. In particular more than 80% of modules contain less than 20 variables. (TIFF) [file pcbi.1002956.s001.tif]

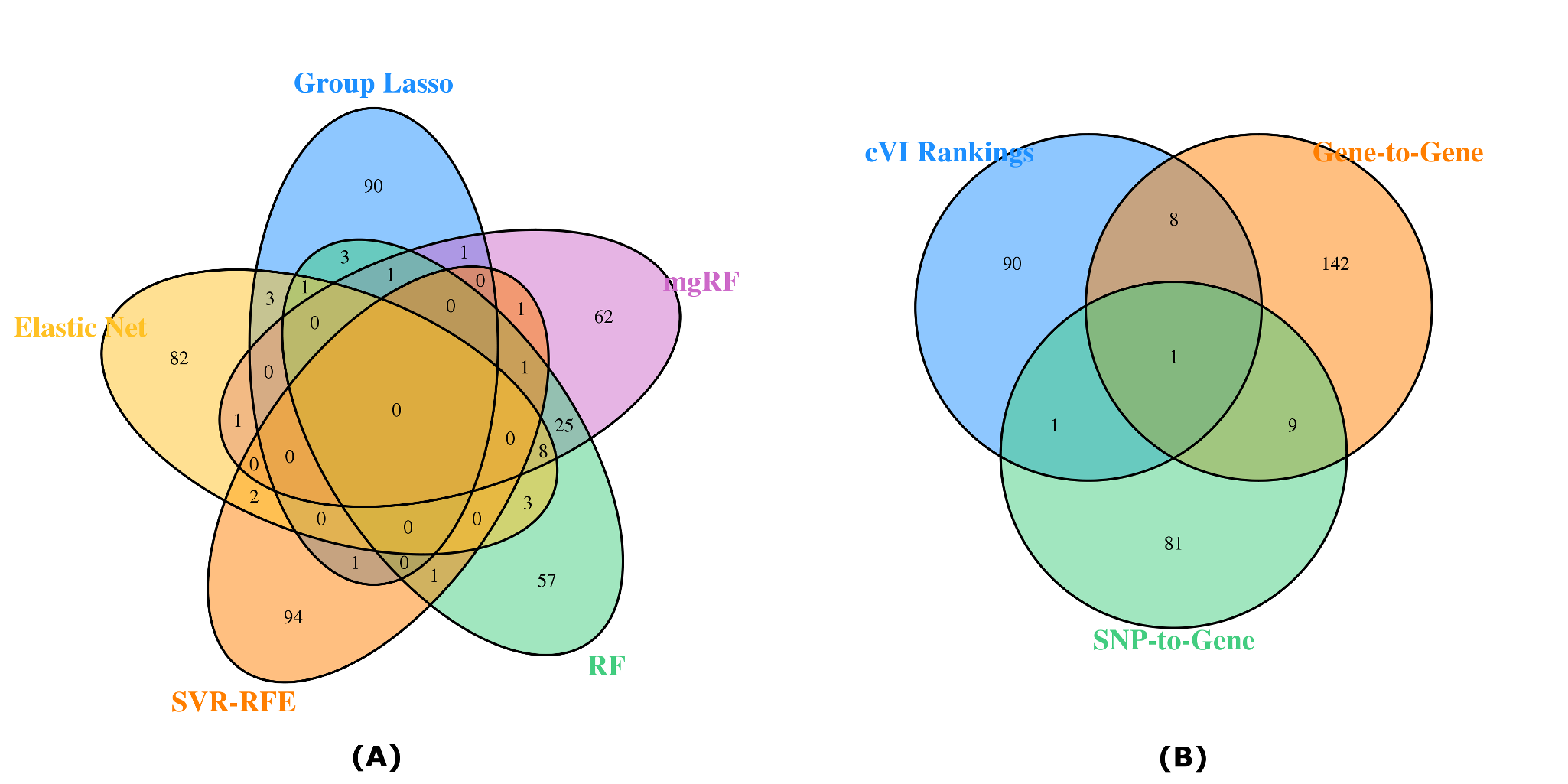

Supplement: Figure S2 — (A) Venn diagram of the 100 top-ranked genes identified by Group lasso (blue), Elastic net (yellow), SVR-RFE (orange), Conventional RF (green), and mgRF (purple). (B) Venn diagram of top ranked genes from gene ranking and genetic element interaction analysis from mgRF. In (B), blue circle represents the top 100 most predictive genes ranked by cVIs. Orange circle represents 160 unique genes ranked in the top 100 most significant gene-to-gene interactions. Green circle represents 100 genes in the top-ranked SNP-to-gene interactions. Most of the genes involved in significant interactions are not individually predictive of mouse weight. (TIFF) [file pcbi.1002956.s002.tif]

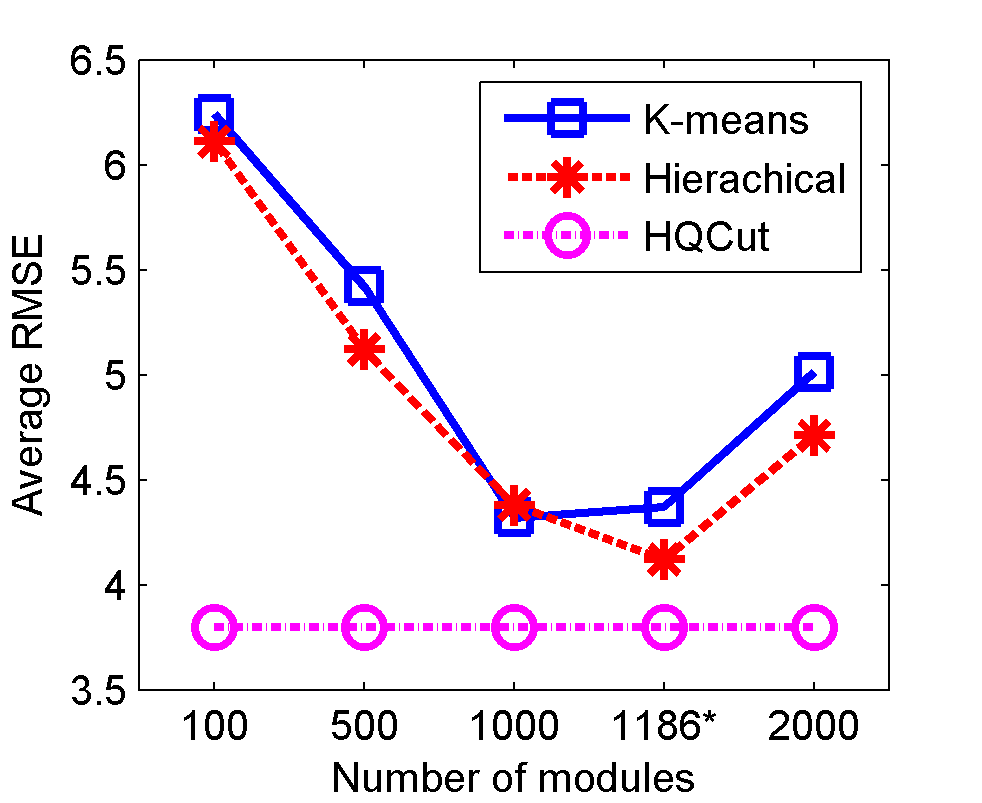

Supplement: Figure S3 — The regression errors of RF and mgRF with different clustering methods and number of clusters. The average RMSEs of mgRF and RF are shown as straight lines because their performance is invariant to the number of clusters. The average RMSEs of other method with respect to a specific number of clusters are shown as (1) Random clustering: dash-dotted line with square markers, (2) K-means: dash line with star markers and (3) Hierarchical clustering: dash-dotted line with cross marker. (TIFF) [file pcbi.1002956.s003.tif]

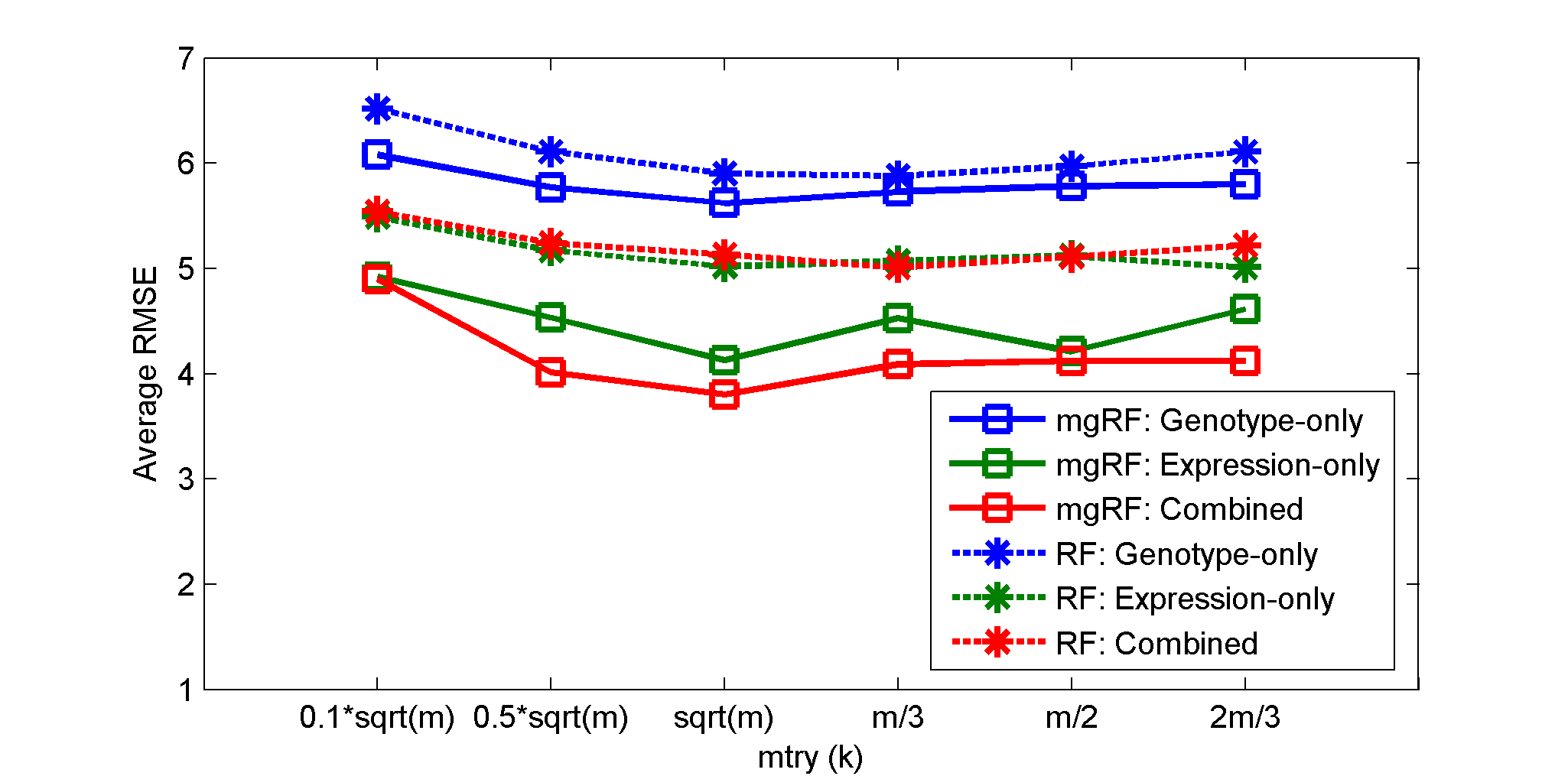

Supplement: Figure S4 — The regression errors of RF and mgRF with respect to different mtry (k) values. The average RMSEs of conventional RF in genotype-only, expression-only, and combined dataset are plotted with dash line and star markers. The average RMSEs of mgRF in genotype-only, expression-only, and combined dataset are plotted with solid line and square markers. (TIFF) [file pcbi.1002956.s004.tif]

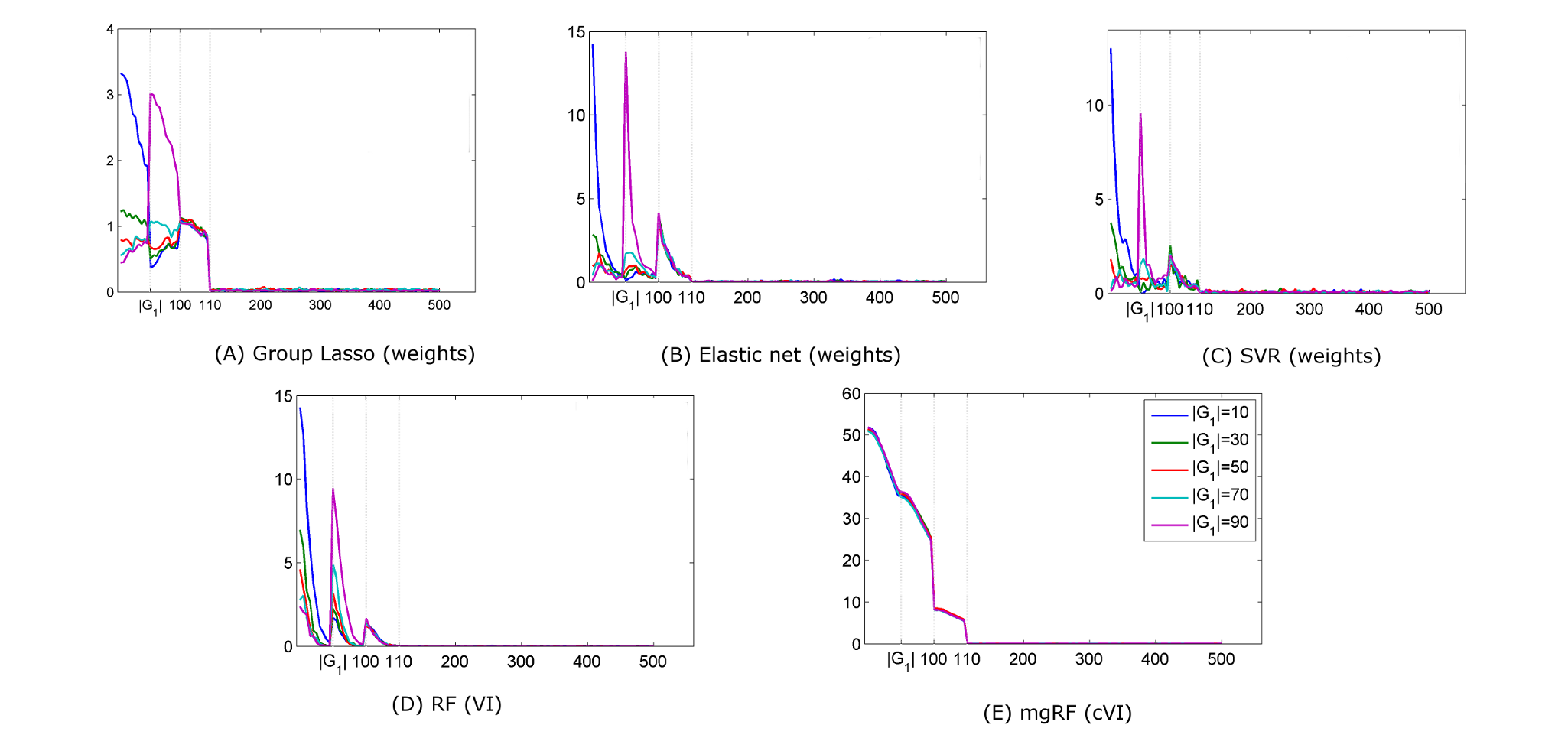

Supplement: Figure S5 — Variable importance identified by various methods with different cardinality of G1 in the simulation dataset. The importance values are normalized to percentage in (A) Group lasso, (B) Elastic net, (C) SVR-RFE, (D) conventional RF, and (E) mgRF. In mgRF, importance values are shown in cVI. The gray dotted lines indicate the grouping of variables. The first 110 variables are relevant variables from construction. For G1 and G2, the importance values of 10 uniformly distributed variables are plotted from each group. (TIFF) [file pcbi.1002956.s005.tif]
