# Supplementary material for: Integrative Analysis Using Module-Guided Random Forests Reveals Correlated Genetic Factors Related to Mouse Weight
Source: PLoS Comput Biol. 2013 Mar 7;9(3):e1002956. doi: 10.1371/journal.pcbi.1002956 (PMC3591263; doi:10.1371/journal.pcbi.1002956)
Supplement: Text S1 — The supplementary text to give the introduction on other regression methods compared with mgRF. (PDF) [file pcbi.1002956.s019.pdf]

Integrative analysis using module-guided Random Forests reveals correlated genetic factors related to mouse weight

Zheng Chen and Weixiong Zhang

Text S1. Other Regression Models

Here we briefly describe the models we evaluated in this work. To compare the variable importance from the following methods, we centered the data at 0 mean and scaled them to 1 standard deviation because the weights of variables are dependent on the scale of variables in those methods.

Group lasso. The l_1-regularized linear regression, a.k.a. lasso [1], is one of the most popular methods for high dimensional regression problems. It models the continuous response as linear functions of input variables. However, with the presence of correlated variables, lasso can only include one or few variables from every group of correlated variables because correlated variables are redundant for the model and will be penalized by the l_1-norm term. Group lasso [2], on the other hand, ensure the sparsity of weight parameters by group, where every group of variables must either be included in the model or be discarded entirety. The variable importance in group lasso is given by:

$$w_{grplasso} = \arg\min_{w} \| y - \sum_{j=1}^{p} X_j w_j \|_2^2 + \lambda \sum_{g=1}^{|G|} \sqrt{d_g} \| w_{I_g} \|_2,$$

where d_g accounts for different group size, I_g indicates the group index of a variable, and $\|.\|_2$ is the Euclidean norm. As $d_g = 1$ for every group g, group lasso reduce to simple lasso. We use the ADMM [3] implementation of group lasso in our experiments.

Elastic net. While enjoying a similar sparsity of representation as in lasso, elastic net also encourages correlated to be grouped together [4]. Unlike group

lasso, the variables grouping structure is not required as input in elastic net and it will attempts to learn it along the way. The variable importance is given by:

$$w_{elasticnet} = \arg\min_{w} w^T \left(\frac{X^T X + \lambda_2 I}{I + \lambda_2} \right) w - 2 y^T X w + \lambda_1 | w |,$$

As $\lambda_2 = 0$, the elastic net reduce to lasso regression. The hyperparameters λ_1 and λ_2 essentially controls the l_1 and l_2 penalty, respectively. We use the *glm_net* [5] implementation of elastic net. λ_1 and λ_2 are all determined by cross-validation.

SVR-RFE. Guyon et al. [6] introduced the recursive feature elimination using support vector machines (SVM-RFE) for gene selection. It has been widely applied to perform feature selection in many biological studies. For regression tasks with continuous response variable, a regression version of SVM, support vector regressor (SVR) can be used. Kernel tricks can also be easily applied to model non-linear response. In the non-linear case, we followed the same procedure introduced in [6], where variable were ranked by their change in cost function caused by removing itself from the data. The changes of cost function for variable v_i is defined as:

$$DJ(vi) = \frac{1}{2} \alpha^T H \alpha - \frac{1}{2} \alpha^T H(-i) \alpha,$$

where α is the learnt SVM dual variables, *H* is the matrix with elements $y_h y_k K(\mathbf{x}_h, \mathbf{x}_k)$, *K* is a kernel function, and the notation (-*i*) means that variable v_i has been removed. In our experiments, we use the radial basis function (RBF) kernel with kernel parameters chosen according to cross-validation.

Reference

1. Tibshirani R (1996) Regression shrinkage and selection via the lasso. Journal of the Royal Statistical Society Series B Methodological 58: 267–288.

2. Yuan M, Lin Y (2006) Model selection and estimation in regression with grouped variables. Journal of the Royal Statistical Society - Series B: Statistical Methodology 68: 49–67.

3. Boyd S (2010) Distributed Optimization and Statistical Learning via the Alternating Direction Method of Multipliers. Foundations and Trends in Machine Learning 3: 1–122.

4. Zou H, Hastie T (2005) Regularization and variable selection via the elastic net. Journal of the Royal Statistical Society - Series B: Statistical Methodology 67: 301–320.

5. Friedman J, Hastie T, Tibshirani R (2010) Regularization paths for generalized linear models via coordinate descent. Journal of Statistical Software 33: 1–22.

6. Guyon I, Weston J, Barnhill S, Vapnik V (2002) Gene Selection for Cancer Classification using Support Vector Machines. Machine Learning 46: 389–422.
