# Supplementary material for: Integrative Analysis Using Module-Guided Random Forests Reveals Correlated Genetic Factors Related to Mouse Weight
Source: PLoS Comput Biol. 2013 Mar 7;9(3):e1002956. doi: 10.1371/journal.pcbi.1002956 (PMC3591263; doi:10.1371/journal.pcbi.1002956)
Supplement: Text S2 — The supplementary text to give the methods and results for several comparisons studies. (PDF) [file pcbi.1002956.s020.pdf]

Integrative analysis using module-guided Random Forests reveals correlated genetic factors related to mouse weight

Zheng Chen and Weixiong Zhang

Text S2. Supplementary Methods and Results

The effects of clustering algorithm on mgRF

In principle, mgRF can take any reasonable clustering result as input as long as the identified module structure groups highly-correlated variables together and separates uncorrelated variables into different modules. However, if variables are randomly grouped into modules, mgRF would assign unnecessary variable importance to unrelated variables such that bias both the *two-stage candidate variable sampling* and the *modified weighted sampling* procedures and eventually leads to low prediction accuracy. Here we assessed the robustness of mgRF under different clustering settings in the combined mouse weight dataset. We applied mgRF with 10-fold cross-validation to obtain the corresponding regression error. For each training folds, we took the output of K-means, Hierarchical clustering, random clustering, and HQCut as the input of the guidance module structure. For K-means, we used the 1 – correlation as the distance measure and iteratively identified given number of clusters. We implemented the Hierarchical clustering in Matlab using the 'linkage' function with the custom distance function as in K-means. For random clustering, given the number of clusters, we randomly permuted the membership of variables from the clustering result of K-means such that their distributions on the number of variables per cluster are the same as in K-means. The results of random clustering were averaged over 20 trails. Note that for HQCut we do not need to specify the number of clusters as it can automatically identify the best number of clusters to optimize the modularity function. Finally we applied conventional RF as a baseline to investigate the benefits of clustering before training. As shown in Figure S3, the RMSE of HQCut is constantly lower than the other clustering settings. When the number of clusters is set to 1180, which is the average number of clusters identified by HQCut, K-means and Hierarchical clustering, both achieves

promising prediction performance. When the number of clusters is close to the 'correct' number, both K-means and Hierarchical clustering outperforms the conventional RF, indicating that mgRF indeed enhances the prediction performance when putative correlated variables are 'correctly' grouped. Random clustering on the other hand provided incorrect information to mgRF and leads to performance degradation.

Sensitivity of mgRF on mtry (k)

The *mtry* (*k*) parameter in the conventional RF algorithm determines how many random variables should be considered at each node of the tree. In mgRF, *mtry* controls the number of modules it random samples at the first stage of candidate variable sampling. A small *mtry* would result in no enough variables being tested and lead to low prediction power. However, a large *mtry* (close to *m, where m* is the total number of variables/modules) would reduce the diversity of RF because at each split it will evaluate almost every possible variables and in turn reduce the effectiveness of RF. Here we set *mtry* to different values (from *0.1*m* to *2*m/3*) and evaluated the 10-fold cross-validation of conventional RF and mgRF on the mouse weight dataset. As shown in Figure S4, our current choice of *mtry=m/3* for RF and *mtry=sqrt*(*m*) achieves satisfactory results in term of average RMSE. In addition, the performance of RF and mgRF are relatively stable when increasing *mtry*. As expected, the error goes up as *mtry* decreases too much (<500).

Simulation Study

We assessed the performance of mgRF on a simulated dataset with known response-relevant variables. With known hidden factors, we aimed to evaluate different methods' ability to recovery the known variable rank. We generated a high-dimensional dataset using a non-linear model similar to a well-known regression problem [1] with correlated variables. The target outcome is given by:

$$y = 0.15\exp(4x_1) + \frac{4}{1+\exp\left(-20(x_2-0.5)\right)} + 3x_3 + \varepsilon \qquad (1)$$

where $\varepsilon \sim N(0, 0.2)$ is a Gaussian random noise. The outcome *y* is associated with three prototype factors: x_1, x_2, and x_2, where $x_i \sim N(0, 1)$ for *i* = 1, 2, and 3. Then we generated three groups G_1, G_2, and G_3, of correlated variables according to x_1, x_2, and x_2 respectively. Variable in each group is given by:

$$v_i^{(j)} = x_i + \left(0.01 + \frac{0.3(j-1)}{n_i - 1}\right) \times N(0, 0.1) \text{ for } j = 1, \ldots, n_i \text{ and } i = 1,2,3 \qquad (2)$$

where $v_i^{(j)}$ indicates the *j*-th variable in group *i* and n_i is the size of group *i*. Apparently the correlation between a variable and its generating factor in each group gradually decreases as the superscript *j* increases. We set the size of third group, n_3 to 10 and the size of second group to n_2 =100-n_1 for different sizes of the first group, n_1 Î $\{10,30,50,70,90\}$ such that we can investigate the effect of varying numbers of correlated variables on their importance measure. We generated 500 variables in total by adding 390 uninformative variables in random group sizes according to (3):

$$v_i^{(j)} = x_i + 0.2 \times N(0, 0.1) \text{ for } j = 1, .. n_i \text{ and } i \neq 1,2,3 \qquad (3)$$

where $x_i \sim N(0, 1)$ and n_i is uniformly distributed from 1 to 10. Similar to the first three groups, uninformative variables are also correlated within group.

We trained models with 100 observations as described above and then tested the models on another 100 observations that were independently generated with the same module structure. We evaluated the performance of variable selection from two aspects. First, we compared the relative variable importance values of different methods to the expected patterns. According to the construction of simulation dataset, we expect to observe decreasing variable importance from the first to the third groups ($VI(v_1^{(j)}) > VI(v_2^{(j)}) > VI(v_2^{(j)})$for the same *j*) and decreasing variable importance within each group ($VI(v_i^{(u)}) > VI(v_i^{(v)})$ for the same *i* when *u* < *v*). For all other groups and variables, we expect to see low importance values. Second, we evaluated the stability of variable importance across different simulations. Although the absolute values of variable importance might change in different simulated dataset due to the noise added to the simulation, the overall patterns of variable importance should be relatively stable. For each method, we quantitatively measured the stability of their variable selection

scheme by averaging all pair-wise Pearson's correlation between variable importance vectors generated from 100 simulations. A stable variable selection method should achieve a high stability score for all variable importance vectors.

Figure S5 summarizes the average importance values of variables assigned by different methods. Since the number of variables in groups 1 and 2 varies, in order to compare patterns of variable importance, we only plotted importance values of 10 uniformly chosen variables in both groups. All models successfully assigned relatively higher importance values to variables in G_1, G_2, and G_3 and low importance to the other variables. The bias of variable importance measure is clearly demonstrated in models other than mgRF. As the cardinality of G_1 increases, importance values of individual variables decreases and conversely, as the cardinality of G_2 decreases, importance values of individual variables increases. In group lasso (Figure S5A), when $|G_1|$=10, the pattern of variable importance is as expected but when $|G_1| > 30$, the relative weights failes to reflect the true pattern of variables importance. For example, when $|G_1| = 90$, most variables in G_1 are falsely assigned to smaller importance values than those in G_2 and G_3. Interestingly, when $|G_1| > 30$, importance value within group increases as the correlation to the hidden factor decreases in both G_1 and G_2. This is probably because the non-linear correlation cannot be successfully modeled by group lasso and random noises increase the linear correlation. Elastic net (Figure S5B) and SVR (Figure S5C) showed similar weights patterns, where the variable importance is relative stable in G_3, but apparently biased when the cardinality of group varied (in G_1 and G_2). In RF (Figure S5D), even though the within-group importance decreases as the correlation to the hidden factor decreases, the ranking of variables given by original VI is incorrect in that when $|G_1| > 50$ ($|G_1|/|G_2| > 1$), variables in G_2 falsely appears to be more relevant than those corresponded in G_1. On the contrary, the cVI in mgRF successfully removed the bias of variable importance. The pattern of importance measure (cVI) in mgRF remains the same regardless varying cardinalities of G_1 and G_2 (shown in Figure S5E).

Table S13 shows the stability score of various methods. mgRF achieves the highest stability score among all settings. SVR and elastic net are the most unstable methods, because usually only one variable from each correlated variable group was assigned a nonzero weight and all others in the same group were given 0 weights. Even

though the data were generated using the same underlying model in each trail, small perturbation on the data (difference among trails) attributed to totally different variables being selected, resulting diverse importance vectors. Group lasso improves the stability a lot compared to elastic net as expected because the correlation structure is given. RF had similar stability pattern to group lasso but has lower stability score as the cardinality of G_1 increases.

Reference

1. Friedman JH (1991) Multivariate adaptive regression splines. Annals of Statistics 19: 1–67. doi:10.1152/japplphysiol.00729.2009.
